# Vascular Injury of Penetrating Trauma of the Extremities

**DOI:** 10.1155/emmi/9979585

**Published:** 2024-12-28

**Authors:** Yeliz Simsek, Aysenur Gur

**Affiliations:** ^1^Department of Emergency Medicine, Adana City Research and Training Hospital, Health Science University, Adana, Türkiye; ^2^Department of Emergency Medicine, Etimesgut Sehit Sait Ertürk Hospital, Ankara, Türkiye

**Keywords:** computed tomography angiography, hard sign, penetrating extremity trauma, soft sign

## Abstract

**Background:** Physical examination and computed tomography angiography (CTA) are used for diagnosing arterial injury in extremity trauma. In recent years, CTA has been overused to obtain more objective data. Our study aimed to investigate the effect of using CTA for the management of patients with extremity penetrating injuries, specifically in cases where vascular injury was not detected during initial examination.

**Methods:** This retrospective study included patients with penetrating trauma who underwent CTA of the extremities. The demographic data, mechanism of injury, the side of injury, initial vascular exam (normal, soft signs, and hard signs), radiological results, and any orthopedic and vascular intervention performed were recorded. The *χ*^2^ test was used for independent variables. A significance level of *p* < 0.05 was used. We compared the sensitivity, specificity, negative predictive value (NPV), and positive predictive value (PPV) for physical exam and CTA for identifying arterial injury requiring intervention.

**Results:** Of the 252 patients included in the study, 29 (21.5%) had abnormal vascular physical examination while 26 (10.3%) had an abnormal CTA. The NPV of the hard sign for identifying vascular injury was 95.4%, while the sensitivity was 57.7%, specificity was 100%, and PPV was 100%. The NPV of routine physical examination to determine the requirement for vascular intervention was 100%. The sensitivity and PPV of the soft sign in determining the need for vascular intervention were 65.4% and 77.3%, respectively.

**Conclusion:** Vascular injury was present in all cases that had positive hard signs. CTA imaging and vascular intervention are not necessary in patients who exhibit no hard and/or soft indicators during a thorough physical examination.

## 1. Introduction

Arterial injuries of the extremities account for almost 50% of all arterial injuries. These injuries can be associated with injuries that pose a risk to the limb and the person's life [1, 2]. Traumatic arterial injury can occur following blunt or penetrating trauma. Penetrating trauma of the extremities (PTE) is associated with a higher incidence of vascular injuries compared with blunt trauma [3].

Physical examination and computed tomography angiography (CTA) are used diagnose patients with PTE vascular injury. According to current trauma guidelines (Eastern Association for the Surgery of Trauma), patients who exhibit certain indicators, known as hard signs, should be immediately taken to the operating room. These signs include the absence of distal pulses, the presence of a growing or pulsatile hematoma, the presence of a murmur or thrill, and presence of active bleeding [4]. A CTA might be requested if there are soft signs, such as decreased pulse, nonpulsatile large hematoma, neurological deficit, excessive bleeding, or for patients at high risk of orthopedic injuries [5].

Recent studies have demonstrated that CTA is being inappropriately utilized for the diagnosis of PTE. Typically, the decision to use CTA imaging is determined based on the institution or the doctor's preference, rather than a standard protocol. The CTA has been excessively used in recent years either to confirm the identification of potential vascular injury or to avoid missing the diagnosis in cases where there is suspicion of vascular injury that cannot be diagnosed through examination. The accessibility of CTA also contributes to this excessive usage [6–10].

In this study, we hypothesize that vascular examination findings might be used to predict vascular injury in PTE and reduce the excessive use of CTA. Our objective was to determine the sensitivity, specificity, positive predictive value (PPV), and negative predictive value (NPV) of physical examination in predicting the requirement for vascular intervention in patients with PTE in the emergency department (ED). Therefore, our aim was to establish a standardized approach to using CTA to determine the need for vascular intervention in PTE based on physical examination findings.

## 2. Patients and Methods

This retrospective observational study was conducted at a single center. Ethical approval was granted by the local ethics committee (No: 139/2936). Patients who were admitted to the ED and underwent CTA of the extremities between January 2022 and December 2022 were scanned using hospital records. Patients with PTE who were age 18 years and older were included in the study.

The exclusion criteria included patients who underwent CTA for nontraumatic reasons, individuals with a history of blunt trauma, and patients who declined treatment. Demographic data (age and gender), clinical details (mechanism of injury, injury side, and vascular physical examination findings), radiological results (type of arterial injuries and fractures), outcomes from the ED (discharge or hospitalization), and information on orthopedic and vascular intervention were collected from electronic hospital records. Mechanism of injury was categorized as either stab wounds and gunshot injuries (GSI). The injury side was classified as either the upper or lower extremity. Vascular physical examination findings were recorded as being within normal limits, and the hard and soft signs. Hard and soft signs are defined and summarized in Table 1 [4, 11].

At our institution, a CTA was requested by the emergency physician as part of the initial trauma evaluation. CTA was performed as a single-phase, single-shot procedure by administering 90–120 mL of contrast material intravenously at a rate of 4 mL/sec using an injector pump and bolus tracking. Intravenous contrast material with a concentration of 350 mg/mL iodine was used for CTA imaging and imaging results were interpreted by the radiologist.

The CTA findings were categorized as either normal or abnormal based on the vascular examination. Vascular injury was assessed by examining active contrast extravasation, aneurysm, occlusion, dissection, vessel wall irregularity, or arteriovenous fistula. Operative intervention was defined as surgical repair, ligation, bypass, intraoperative arteriography, or amputation.

### 2.1. Statistical Analysis

The study data were analyzed using IBM SPSS Statistics 29.0 (Statistical Package for the Social Sciences–IBM) software. Mean, standard deviation, and percentage values were calculated for the descriptive data. The *χ*^2^ test was used to examine the relationships between independent variables. A significance level of *p* < 0.05 was used for all tests. Vascular injury was defined as an abnormality detected using CTA that required vascular intervention. The sensitivity, specificity, NPV, and PPV of the hard sign, soft sign, and normal physical examination were calculated for identifying the arterial injury.

## 3. Results

A total of 674 patients had CTA of extremities within 1 year. 252 patients who met the inclusion criteria were enrolled in our study. The mean age of the patients was 33.45 ± 10.842 and the males accounted for 96.4% of the total cohort. The mechanism of injury, the side of the injury, findings of vascular physical examination, radiological results, and any surgical interventions performed (orthopedic or vascular) on the patients are summarized in Table 2. 89.7% of all patients had lower extremity injuries, with the most common injury being GSI (65.5%). The vascular physical examination was normal in 223 (88.5%) patients. There were 29 (21.5%) patients with abnormal physical examinations. Seven patients had only hard signs while 14 had only soft signs. The CTA results of 26 (10.3%) patients were abnormal and vascular surgery was performed in 26 (10.3%) patients.

A significant relationship was found between CTA and the presence of hard signs (*p* < 0.001) and soft signs (*p* < 0.001). The CTA was abnormal in 26 patients. Of the 26 patients with positive CTA, 15 had hard signs while 11 had soft signs. Three patients who had abnormal physical examinations and negative CTA results had only soft sign findings on examination and did not require vascular intervention.  Table 3 provides a summary of the sensitivity, specificity, NPV, and PPV of the physical examination for vascular injury, especially for hard signs, soft signs, and normal findings.

While the sensitivity of the hard signs for detecting vascular injury was 57.7%, its specificity was 100%. The presence of vascular injury was detected in all cases that had positive hard signs (PPV = 100%). However, the absence of a hard sign did not exclude vascular injury (NPV = 95.4% and sensitivity = 57.7%). All patients who did not show any hard and/or soft signs on physical examination had normal CTA and did not require vascular intervention (NPV = 100%). The sensitivity of soft signs in determining the need for vascular intervention was calculated as 65.4% and the PPV was 77.3%.

According to our data, a statistically significant relationship was found between the injury side and the presence of a hard sign (*p* < 0.001) or soft sign (*p*=0.001), CTA (*p* < 0.001), and the need for vascular surgery (*p* < 0.001). The incidence of vascular injuries and the requirement for vascular intervention were greater in upper extremity injuries compared with lower extremity injuries. There was no significant relationship between the type of injury and the requirement for vascular intervention (*p*=0.188).

70 (27.8%) patients had fractures caused by trauma and 41 (58.6%) of them underwent orthopedic surgery. There was no statistically significant relationship between the presence of a hard sign and the occurrence of a fracture (*p*=0.593) or the requirement for orthopedic surgery (*p*=0.547). There was no statistically significant relationship between the presence of a fracture and the necessity for vascular intervention (*p*=0.438). Fractures were more common with GSI (*p* < 0.001).

## 4. Discussion

Penetrating extremity traumas are associated with vascular injuries [3]. In patients with PTE, a rapid and accurate diagnosis in the ED along with the planning of appropriate treatment is crucial for preserving both the affected limb and the patient's life. Physical examination and CTA are used for evaluation of vascular injuries and both allow for effective patient care. Comprehensive physical examination and appropriately requested CTA might reduce the necessity for hospitalization or prolonged observation periods. However, studies show that CTA is overused [12]. Arguably, the primary factor contributing to this overuse is its convenient availability. It is important to remember that the excessive and unnecessary use of CTA causes an increase in adverse effects resulting from radiation and exposure to contrast material. In a study by Roux, Plessis, and Pitcher arterial injury was found in 10% of patients with lower extremity penetrating trauma. There was an average annual increase of 13% in the number of CTAs performed, but there was no significant change in the rate of CTAs showing arterial injury [13].

Similar to our study, others have shown that the decision to schedule a CTA depended on the specific institution or physician suggesting no standardized protocol for requesting CTA. Kelly et al., found that the sensitivity of CTA in determining the need for vascular surgery was 100%, with a specificity of 58.6%, and PPV of 23.1% [12]. Seamon et al. indicated the sensitivity and specificity of CTA in detecting vascular injury in PTE as 100% [14]. In our study, all patients with abnormal findings on CTA underwent vascular surgery. Our study found that the sensitivity, specificity, PPV, and NPV of CTA in evaluating the necessity for vascular intervention were all 100%.

Assessing vascular physical examination findings (known as hard and soft signs) is crucial for the management of patients with PTE and for determining the need for CTA. The identification of a hard sign on physical examination typically indicates the need for immediate vascular intervention without the use of imaging techniques [12, 15]. In certain instances of injuries that are hemodynamically stable and have hard signs, preoperative CTA can be used to explore other treatment options (eg, stent or embolization) instead of resorting to unnecessary vascular surgery [16, 17]. Warwick et al. discovered that the accuracy of predicting the need for vascular intervention in wounds of extremities was higher using hard signs compared with using CTA, (PPV 35% vs. 50%, respectively) [7]. In our study, the sensitivity of hard signs for detecting vascular injury was 57.7% and its specificity was 100%. In all cases where hard signs were present, there was evidence of vascular damage (PPV = 100%, NPV = 95.4%, and sensitivity = 57.7%).

CTA is recommended to establish the need for surgery in patients with a soft sign or in patients with a diagnosis cannot be made by a vascular physical examination [7, 13, 14, 18]. In the study by Gurien et al., of 114 patients with PTE without hard signs who underwent CTA, soft signs were identified in 29 of the patients (25.4%). Furthermore, hard signs were detected in three of four patients where the first examination was normal but CTA was positive in a repeat examination. Based on their findings, the use of CTA only improved the diagnostic accuracy by 0.5% in patients who showed no clear signs of injury but had a correct physical examination [9]. Our investigation found that the CTA results were normal for three individuals who had abnormal physical examinations and none of them required vascular intervention. There was a soft sign in the examination findings of these three patients. We found that the sensitivity of soft signs in determining the need for vascular intervention was 65.4% and its PPV was 77.3%.

Even if the vascular physical examination is normal, CTA is conducted as a precautionary measure to avoid missing the diagnosis and to investigate the possibility of vascular injury. However, most studies emphasize that performing a CTA is not required during a routine physical examination. Kelly et al. discovered that the NPV of a normal physical examination in detecting vascular injury was 100% with a sensitivity of 100% [12]. The study by Callan, Bauer, and Mir, included individuals who experienced both blunt and penetrating injuries to their extremities. Of the 117 patients who had normal physical examination findings, there was one patient with an abnormal CTA (femoral artery injury) who underwent surgical vascular intervention [19]. The study by Joseph et al. found that the CTA results of all patients who had normal physical examinations with lower extremity trauma were also normal. Vascular intervention was not performed in any patient with a negative CTA [6]. In the study by Warwick et al., all 22 patients who had normal physical examinations but abnormal CTA did not prompt the need to undergo vascular surgery (NPV = 100%). The CTA abnormality was caused by compartment syndrome or edema resulting from blunt trauma [7]. Our study exclusively focused on penetrating traumas, and we did not observe any patients with a normal physical examination who had an abnormal CTA. The sensitivity and NPV of a normal physical examination in determining the necessity for vascular intervention were both 100%. According to our study, if the physical examination shows normal vascular conditions, there is no need for CTA or vascular intervention.

In this study, similar to previously published studies, we found that PTE predominantly affects men and is primarily caused by GSI [6, 7, 9, 12, 13, 19, 20]. We found no difference between stabbing wounds and GSI in terms of causing vascular injury. We also found that fractures were more common in patients with GSI. According to our study, 27.8% of the patients had a fracture and 58.6% of them needed orthopedic surgery. However, there was no significant difference in the requirement for vascular intervention among those with fractures or those who underwent orthopedic surgery because of fractures. Similar to our study, Rouz, Plessis, and Pitcher discovered that the occurrence of fracture in penetrating lower extremity trauma had no correlation with arterial injury [13].

### 4.1. Limitation

An important limitation of our study was its retrospective nature. We depended on medical records for physical examination findings. We lacked data on patients with PTE who did not undergo CTA.

## 5. Conclusion

The incidence of PTE was predominantly observed in men and the mechanism of injury was mostly GSI. No relationship was found between the mechanism of injury or the presence of fracture and vascular injury. Vascular injury was present in all cases that showed positive hard signs. Patients with soft sign results might have a risk of vascular injury, therefore, it is recommended to perform a CTA in these cases. CTA imaging and vascular intervention are not necessary in patients who exhibit no hard signs and/or soft signs upon a thorough physical examination. Prospective multicenter studies are needed to evaluate the validity of physical examination in predicting vascular injuries and to develop algorithms to prevent CTA overuse.

## Figures and Tables

**Table 1:** Vascular physical exam classification definitions.

Soft signs	Diminished pulse, neurologic finding from nerve adjacent to a named artery, or a small non-pulsatile hematoma over a named artery
Hard signs	Pulselessness, rapidly expanding hematoma, massive bleeding, or palpable or audible bruit
Normal vascular exam	Normal pulse, warm and perfused distal extremity

**Table 2:** Summary of mechanism of injury, physical examination, and imaging findings, type of operation.

	Number of patient (*n*)	Percentile (%)
Injury side		
Upper extremity	26	10.3
Lower extremity	226	89.7
Mechanism of injury		
Gunshot injury	165	65.5
Stab injury	87	34.5
Hard sign		
No	237	94
Yes	15	6
Soft sign		
No	230	91.3
Yes	22	8.7
Fracture		
No	182	72.2
Yes	70	27.8
CT angiography		
Abnormal	26	10.3
Vascular operation		
No	226	89.7
Yes	26	10.3
Orthopedic operation		
No	211	83.7
Yes	41	16.3
Total number of patient	252	100

**Table 3:** Sensitivity, specificity, negative predictive value (NPV), positive predictive value (PPV) of hard sign, soft sign, normal physical examination for vascular injury requiring intervention.

	Sensitivity (%)	Specificity (%)	NPV (%)	PPV (%)
Soft sign	65.4	97.8	96	77.3
Hard sign	57.7	100	95.4	100
Normal physical examination	100	97.7	100	83.9

## Data Availability

Data are available on request due to privacy/ethical restrictions.
